# Caudal Nucleus Accumbens Core Is Critical in the Regulation of Cue-Elicited Approach-Avoidance Decisions

**DOI:** 10.1523/ENEURO.0330-16.2017

**Published:** 2017-02-15

**Authors:** Laurie Hamel, Tharshika Thangarasa, Osai Samadi, Rutsuko Ito

**Affiliations:** 1 Department of Psychology (Scarborough), University of Toronto, 1265 Military Trail, Toronto, Ontario M1C 1A4, Canada; 2 Department of Cell and Systems Biology, Ramsay Wright Laboratories, University of Toronto, 25 Harbord Street, Toronto, Ontario M5S 3G5, Canada

**Keywords:** conditioned approach, conditioned avoidance, motivation, NAc, reward, punishment

## Abstract

The nucleus accumbens (NAc) is thought to be a site of integration of positively and negatively valenced information and action selection. Functional differentiation in valence processing has previously been found along the rostrocaudal axis of the shell region of the NAc in assessments of unconditioned motivation. Given that the core region of the NAc has been implicated in the elicitation of motivated behavior in response to conditioned cues, we sought to assess the role of caudal, intermediate, and rostral sites within this subregion in cue-elicited approach-avoidance decisions. Rats were trained to associate visuo-tactile cues with appetitive, aversive, and neutral outcomes. Following the successful acquisition of the cue-outcome associations, rats received microinfusions of GABA_A_ and GABA_B_ receptor agonists (muscimol/baclofen) or saline into the caudal, intermediate, or rostral NAc core and were then exposed to a superimposition of appetitively and aversively valenced cues versus neutral cues in a “conflict test,” as well as to the appetitive versus neutral cues, and aversive cues versus neutral cues, in separate conditioned preference/avoidance tests. Disruption of activity in the intermediate to caudal parts of the NAc core resulted in a robust avoidance bias in response to motivationally conflicting cues, as well as a potentiated avoidance of aversive cues as compared with control animals, coupled with an attenuated conditioned preference for the appetitive cue. These results suggest that the caudal NAc core may have the capacity to exert bidirectional control over appetitively and aversively motivated responses to valence signals.

## Visual Abstract

**Figure:**
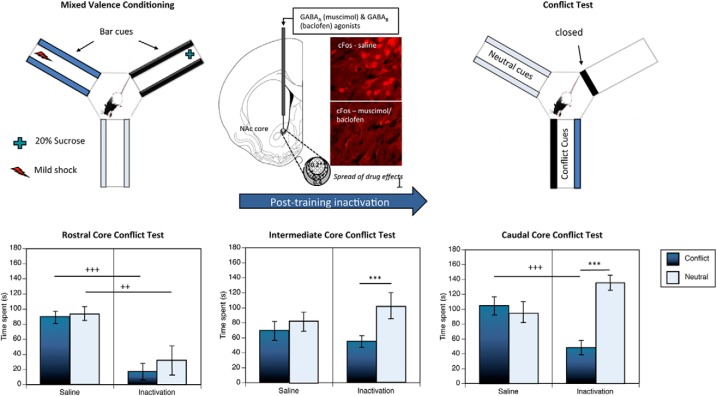

## Significance Statement

The nucleus accumbens (NAc) is a heterogeneous structure, known as a site of confluence of limbic information. Although traditionally thought to divide into shell and core subregions, recent evidence has demonstrated a topographical organization of valence information processing along a rostrocaudal gradient in the shell. However, such rostrocaudal differentiation of valenced information has not been well characterised in the core. Using transient, localized inactivation of the core combined with a novel approach-avoidance conflict paradigm, we found evidence of a dissociation between the rostral and caudal core in the processing of valenced cues in motivating approach and avoidance responses. Our findings implicate the caudal core to be critical in facilitating cued approach, while suppressing cued avoidance in the face of motivational conflict.

## Introduction

The resolution of approach and avoidance decisions in response to environmental stimuli is one of the most fundamental adaptive processes required for species survival and reproduction. In mammals, evaluative and motivational functions are instantiated in a network of brain structures including the hypothalamus, neocortical regions, and limbic sites such as the hippocampus, amygdala, and nucleus accumbens (NAc) (Balleine and Dickinson, 1998; Cardinal et al., 2002; Pennartz et al., 2011; Gruber and McDonald, 2012; Nieh et al., 2013). The NAc is a structure of particular interest given that it is a site of confluence in the processing of valenced information from these other regions and has efferent connections to motor output structures. As such, it has been considered as a limbic-motor interface functionally linking motivation and action (Mogenson et al., 1980). Although the NAc has traditionally been implicated in mediating reward-directed behavior, evidence also suggests that it plays a role in the evaluation of aversive or threatening stimuli (Roitman et al., 2005; Carlezon and Thomas, 2009), and there is an increasing emphasis on viewing this structure as a site of integration of both positively and negatively valenced information (Levita et al., 2009; McCutcheon et al., 2012). It is therefore proposed to be a key site where this bivalent information is used in the selection and elicitation of appropriately directed behavior (Humphries and Prescott, 2010).

The NAc has been divided into separate subregions, core and shell, based on studies indicating differential morphology, histochemistry, connectivity, and function (Voorn et al., 1989; Meredith et al., 2008). The NAc shell has been posited to mediate the primary or unconditioned rewarding effects of drugs and natural reinforcers (Carlezon et al., 1995; Pontieri et al., 1995; Ito et al., 2000, 2004; Aragona et al., 2008), whereas the NAc core has often been implicated in mediating the motivation or elicitation of instrumental and approach behaviors in response to reward-associated cues (Parkinson et al., 2000; Ito et al., 2004; Stefanik et al., 2013). Beyond the delineation of core and shell, however, there is increasing evidence of a physiological and functional differentiation arranged topographically within these boundaries. Studies have indicated the existence of a rostrocaudal gradient in the processing of valence within the shell; that is, that glutamate receptor antagonism or GABA_A_ receptor agonism in the rostral shell leads to increases in appetitive behavior, whereas neural disruption in the caudal shell leads to aversively biased behavior. This effect has been observed in the expression of food consumption (Sratford et al., 1998; Reynolds and Berridge, 2001), hedonic taste reactions (Reynolds and Berridge 2002; Faure et al., 2010), innate defensive behaviors (Reynolds and Berridge, 2003), and conditioned place preference (Reynolds and Berridge, 2002).

The NAc core has been less fully characterized in terms of a potential gradient in valence processing as compared with the shell. A study by Reynolds and Berridge (2003) failed to find a rostrocaudal gradient in the NAc core in unconditioned fear or feeding behaviors as elicited by glutamate receptor antagonist microinfusions. However, the effects of rostrocaudally differentiated GABA receptor manipulations within the NAc core on bivalent motivated behavior have yet to be assessed. Furthermore, the rostrocaudal differentiation in the core has not been investigated in relation to conditioned motivated behavior, which is an important undertaking given the well-established role of the core in cue-elicited motivated behavior (Ito et al., 2004; Stuber et al., 2011; Stefanik et al., 2013). Thus, in the present study, we sought to determine whether localized disruption of core function may yield differential effects on cue-elicited approach or avoidance behavior along the rostrocaudal axis, using a novel concurrent conditioned cue preference and avoidance paradigm. We found that GABAR-mediated disruption of activity in more caudal aspects of the NAc core resulted in an avoidance bias toward motivationally bivalent (conflicting) cues, as well as a potentiation of conditioned cue avoidance, and an attenuation of conditioned cue preference. Our results therefore indicate that the NAc caudal core is critical in the expression of approach-avoidance behavior elicited by cues of positive and negative valence.

## Materials and Methods

### Subjects

Experimental procedures were performed using 75 male Long-Evans rats (Charles River Laboratories, RRID:RGD_2308852) weighing between 350 and 400 g at the time of surgery. They were housed in pairs under a 12/12 h light/dark cycle, with lights turning off at 7:00 P.M. Experiments occurred during the light phase of the cycle. Water was available ad libitum, but 2 d before the start of behavioral testing, food was restricted to sufficiently maintain their body weight at 85% of their free feeding weights. All animal procedures were performed in accordance with the University of Toronto animal care committee's regulations.

### Surgery

Animals were anaesthetized using 3-4% isofluorane and placed in a frame for stereotaxic surgery. A 26-gauge stainless steel bilateral guide cannula (Plastics One) was implanted into one of the following coordinates (in mm from bregma): rostral accumbens core (AP = +1.7, ML = ±1.5, DV = -5.6), intermediate accumbens core (AP = +1.2, ML = ±1.5, DV = -5.7), or caudal accumbens core (AP = +0.7, ML = ±1.5, DV = -5.7). At the time of microinfusions, injector tips extended by 1 mm below the guide cannulae, and thus the final targeted location was at 1 mm ventral to the above coordinates. The guide cannulae were affixed to the skull using dental cement and jeweler’s screws. Stainless steel stylets were inserted into the guide cannulae to maintain patency. Rats received injections of 5 mg/kg ketoprofen, 20 min before awakening as an analgesic. Animals underwent a minimum recovery period of 7 d in their homes cages before beginning experimental training.

### Experimental procedures

#### Apparatus

**Figure 1.**
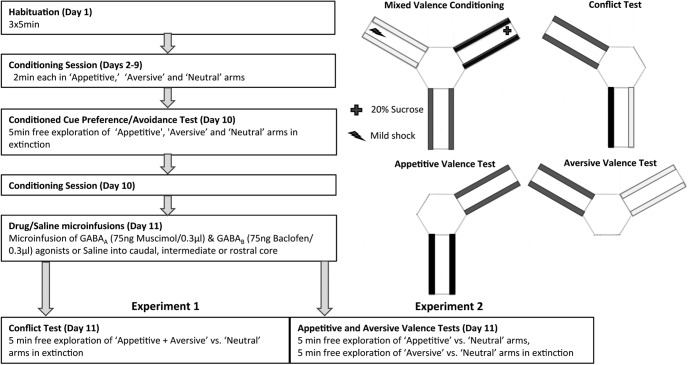
Schematic diagram showing the sequence of events (left) and the apparatus and cues used (right) in the novel concurrent mixed valence conditioning paradigm for rats. Animals were trained to associate three different cues (bar inserts) with an appetitive outcome (sucrose), aversive outcome (mild shock), or no outcome (neutral) in eight conditioning sessions. The acquisition of cue-outcome associations was then assessed by a conditioned cue preference/avoidance test (in extinction), followed immediately by one further conditioning session. The following day, animals underwent a “conflict test” (experiment 1), in which they were simultaneously presented with an aversive and appetitive cue within one arm (conflict arm), and the neutral cue in the other arm. The time spent in the “conflict” arm was compared with the time spent in the neutral arm. In experiment 2, rats underwent two separate tests of “appetitive valence” and “aversive valence” (in counterbalanced order). In these tests, conditioned preference for the appetitive cue (over neutral cue) and conditioned avoidance of the aversive cue (over neutral cue) were measured.

Behavioral training and testing were conducted using a six-arm radial maze apparatus (Med Associates). The six arms converged at a hexagonal hub where automated steel guillotine doors controlled access into each arm [45.7 cm (L), 9 cm (W), 16.5 cm (H)]. The arms were enclosed with Plexiglas walls, a removable lid and a steel grid floor, which was connected to a footshock-generating device (Med Associates). A receding well was located at the end of each arm, which was connected to a polythene tubing and a syringe for the delivery of sucrose solution. The entire apparatus was covered with red cellophane to obscure extramaze stimuli. Med PC IV software was used to control the timing of maze door opening. A ceiling-mounted camera was positioned above the apparatus to allow for monitoring and recording of test sessions. Sessions occurred under illuminated conditions to ensure cue visibility. Only three out of six arms (forming a Y maze, see Figure 1) were used at any one time in an experimental session.

#### Cues

During training and test trials, wooden rectangular inserts measuring 45 × 2.5 cm and covered with either gray duct-tape, blue denim cloth material, or exposed wooden finish were placed along the lower half of the walls of the maze arms and affixed using Velcro. The inserts were thus discriminable in terms of texture, color, and reflective properties. The inserts became cues predictive of sucrose availability, mild footshock administration, or neutral conditions (no scheduled events) in a given arm during the training sessions. The valence of cues for a given rat was determined following the unvalenced cue habituation session (described below), whereby any innate preference for a cue was counterbalanced by assigning it to the opposite valence during conditioning.

### Behavioral training procedures (Fig. 1)

#### Habituation (d 1)

Three habituation sessions were conducted. During the first habituation session, no cues were inserted into the arms. Rats were placed individually in the central hub to begin the session with all doors closed. After one minute, three doors were raised to allow free exploration of the identical arms and the hub for a period of 5 min. After this time, all doors were lowered, and the animal was removed from the apparatus. During the second habituation session, the cues were inserted into 3 separate arms, and the same sequence of events occurred as above. The amount of time spent exploring each arm was measured and used to determine the valence of each cue for a given rat, as explained above. During the third habituation session, animals were presented with two sets of cues inserted into two arms; the cues assigned as the “neutral” cues in one arm, and a combinatorial cue in the other arm, consisting of one bar of different texture and color on either side of the arm, to mirror the conditions of the final conflict test. This session was crucial in ensuring that the combination of stimuli presented in the conflict test will not be novel to the animals. Following the habituation sessions, rats were given access to sucrose solution in their home cages for 5 min to habituate to its consumption.

#### Mixed valence conditioning training (d 2-9)

Training sessions were conducted once daily over a period of eight consecutive days. The appetitive, aversive, and neutral cues were placed in randomized arms before each session, varied between subjects within a session, and within subjects between sessions, to minimize any conditioning to extraneous intra-maze cues or odors. A syringe for sucrose administration was connected via polyethylene tubing to the well in the arm containing the appetitively valenced cue. The flooring in the arm containing the aversively valenced cue was connected to the footshock generator. At the start of each session, a rat was confined in the central hub. After 30 s, a door was elevated to allow access to one arm. On entry of the animal, the door was lowered to restrict the rat to that arm for a period of 120 s. During this time, the animal was administered either the unconditioned appetitive, aversive, or neutral stimulus, depending on the intended valence of the cue contained within that arm. The appetitive unconditioned stimulus was administered as an infusion of 0.2 ml aliquots of 20% sucrose solution delivered four times at an interval of ∼30 s. The aversive unconditioned stimulus was a footshock lasting 0.5 s of ∼0.25 mA (mild shock), calibrated to a level at which the animal demonstrated a startle response, and not a freezing response. Four footshocks were administered at ∼30 s intervals. Extensive piloting was conducted to optimize the magnitudes of the sucrose reward and shock required to facilitate the acquisition of conditioned approach and avoidance respectively, while preventing the induction of generalized fear of the whole apparatus, and the development of freezing responses to the cue paired with shock (see Ito and Lee, 2016). Furthermore, the optimized magnitudes of the sucrose solution and shock served to ensure that the acquired positive and negative incentive values of the cues were balanced (and not one dominating over the other) so as to eliminate any preference for, or avoidance of the jointly presented positive and negative cues in the control subjects during the conflict test. This way, any significant deviations from this “norm” (increased approach or avoidance of the conflict cue) could be easily detected.

At the end of the 120 s period in each arm, the door was opened to allow reentry of the animal into the central hub, whereupon it was confined for 30 s until a second arm was opened. The same procedure then followed for the cued arms of the other 2 valences, and the animal was subsequently removed from the apparatus to terminate the session. The order of entry into arms of each valence was varied across sessions. The arms were cleaned with 70% ethanol solution between animals and sessions. The maze was rotated by varying degrees (60°, 120°, 180°) at the end of each testing day to prevent any conditioning to extramaze spatial or contextual cues.

#### Conditioned cue preference and avoidance test (d 10)

To assess the acquisition of conditioned cue preference/avoidance, a test session was conducted under drug-free conditions on d 10. The test session followed the protocol used during the second habituation session. The rat was given 5 min to freely explore the three arms, which each contained one of the three sets of valenced cues, but no unconditioned stimuli were presented. The amount of time spent exploring each arm was measured and recorded via video monitoring. A rat was determined to have entered a given arm when both its front and hind paws crossed the door threshold, and likewise to have exited the arm when all of its paws had entered the central hub. The conditioned cue preference/avoidance test was immediately followed by an additional session of conditioning on the same day.

### Drugs and microinfusions

For 3 d before the drug infusion sessions, animals were habituated to gentle hand restraint in the manner and environment in which infusions were to be administered. On the day before the first drug session, all animals received an infusion of the saline vehicle, to minimize the mechanical effects of subsequent infusions and to further habituate the animals to the procedure. On infusion days, animals received 0.3-μl bilateral intracerebral microinjections of a solution containing a mixture of the GABA_A_ receptor agonist muscimol and the GABA_B_ receptor agonist baclofen (75 ng of each drug per infusion; Sigma-Aldrich) dissolved in physiological saline, or the saline vehicle only. The drug was infused via 33-gauge microinjectors projecting 1 mm below the indwelling guide cannulae using an infusion pump (Harvard Apparatus) mounted with 5-μl Hamilton syringes. The infusion occurred at a rate of 0.3 μl/46 s, and the injector was left in place for an additional 1 min to ensure complete diffusion of the drug from the injector tip. Approximately 10-15 min after the end of each infusion, the conflict test, or appetitive/aversive valence tests, was administered (described below).

### Tests

#### Experiment 1: conflict test for the expression of approach versus avoidance decisions

A test session was conducted following the drug or saline microinfusions into the caudal, intermediate, or rostral NAc core on d 11. In this test, rats were presented with two arms (instead of three): one in which the aversively conditioned cue was superimposed with the presentation of the appetitive cue within a single arm and another in which the neutral cue was placed. The rat was allowed to explore both arms in addition to the central hub for 5 min. No unconditioned stimuli were administered. The amount of time spent exploring each arm was measured and recorded via video monitoring, to indicate decision-making biased toward approach tendency (increased full entries and time spent in the conflict over neutral arm) or avoidance tendency (decreased entry into and time spent in the conflict arm) in the face of motivational conflict.

#### Experiment 2: separate appetitive and aversive valence tests

Additional conditioned preference/avoidance tests were conducted in a separate cohort of rats (*n* = 22) that had received cue training in an identical manner, to assess potential differences in motivated approach and avoidance behavior in response to separate aversive and appetitive cues. Following drug/saline microinfusions into the intermediate/caudal NAc core, rats were free to explore the central hub and radial maze for 5 min, wherein one arm contained the neutral cue, and a second arm contained either the aversive or appetitive cue. Each rat underwent both tests, with the order of the testing counterbalanced. Exploration time was measured under extinction conditions.

### Elevated plus maze

An elevated plus maze task was used to measure unconditioned anxiety levels in a subgroup of animals undergoing saline-infusions or inactivation of the intermediate or caudal NAc core (saline, *n* = 11; inactivation, *n* = 9). The task was performed in a plus maze that was placed in a novel room and was elevated 50 cm from the floor. It contained a central platform [10 cm (L) × 10 cm (W)] that connected four arms [40 cm (L) × 10 cm (W) × 22 cm (H)], with two open arms and two arms that were enclosed by walls (closed arms). The rat was placed in the central compartment of the maze facing an open arm at the start of the session and was thereafter allowed to explore the maze for 10 min. The entries into open and closed arms as well as the time spent in the arms were measured.

### c-Fos characterization of the spread of drug effects

A separate group of rats (*n* = 8; *n* = 4 each in drug versus saline groups) was used for the assessment of the distance of effective spread of baclofen/muscimol microinfused into the intermediate or caudal NAc core region, as evidenced by changes in c-Fos activity near the injection site compared with saline vehicle controls. c-Fos expression was labeled by immunohistochemistry and visualized by immunofluorescence. Rats were perfused transcardially 75 min after bilateral microinjections of 0.3-μl saline containing 75 ng of both baclofen and muscimol, or the saline vehicle alone into intermediate-caudal NAc core. Extracted brains were placed in 4% PFA overnight. Brains were then cut in 50-μm slices using a vibratome (Leica) and stored in 60% glycerol in 0.1 M PBS containing 0.1% sodium azide. During immunofluorescence processing, brain slices were gently agitated in 3 successive washes with PBS, then incubated in 10% normal goat serum (NGS) blocking solution for 1 h at room temperature. Slices were subsequently incubated overnight at 4°C in rabbit anti-c-Fos (Santa Cruz Biotechnology catalog #sc-52, RRID:AB_2106783) at 1:500 in 5% NGS and 0.1% Triton X-100 in PBS. The following day, slices were washed 3 times with PBS in 10-min intervals and were then incubated for 2 h while light restricted at room temperature in a Rhodamine (TRITC) AffiniPure goat anti-rabbit IgG (Jackson ImmunoResearch Labs) and Hoescht Dye in 5% NGS and 0.1% Triton X-100 in PBS. Slices were subsequently washed 3 times with PBS in 10-min intervals, then mounted onto slides and coverslipped using Aqua Polymount and stored protected from light at 4°C.

c-Fos-like immunoreactivity was visualized at 20× magnification using a Nikon microscope equipped for fluorescence microscopy. c-Fos-labeled cells were individually counted in sample quadrants of the annuli formed at 0.10-mm intervals of a radial grid centred at the microinjection site.

### Data analysis

All data were analyzed using the SPSS statistical package version 23.0 (SPSS). To assess animals’ learning of the valenced cue associations during the training period, a three-way ANOVA was conducted for the dependent measure of time spent in each arm (appetitive, neutral, aversive), with within subject factors of future drug group (saline versus baclofen/muscimol) and cannula location (rostral, intermediate, or caudal core) to determine whether there were any preexisting group differences in learning. To assess motivational bias during the conflict test, a three-way ANOVA was performed with within subjects factors of drug group (saline versus baclofen/muscimol) and location of the microinfusion (rostral, intermediate, or caudal accumbens core). The dependent measure was the amount of time spent in the conflict arm versus the time spent in the neutral arm. Where there was a significant violation of homogeneity of variance across groups for a repeated measures design, as assessed by Mauchly’s test of sphericity, the Greenhouse-Geisser ε was used to calculate a more conservative *p* value for each *F* ratio. Furthermore, significant main within subject effects, thee-way or two-way interactions were further explored using simple effect analyses and planned/*post hoc* comparisons (performed with Bonferroni correction).

### Histology

After completion of the behavioral testing, animals were sacrificed using 1200-mg/kg chloral hydrate (Sigma-Aldrich) and perfused intracardially with 100-ml saline, followed by 100 ml of 4% paraformaldehyde (PFA) in PBS. Brains were then removed and stored in PFA before being transferred to a sucrose cryoprotectant. Coronal slices of 50 μm in diameter were cut with a freezing microtome and then stained with cresyl violet for viewing under a microscope to verify the placement of cannulae.

## Results

### Cannula placement

**Figure 2.**
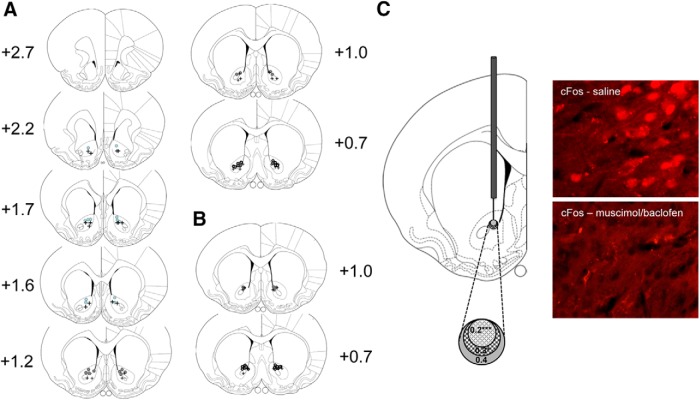
***A***, Schematic representation of the locations of injector tips in the NAc core rostral (AP coordinates +2.2, +1.7, +1.6), intermediate (AP coordinates +1.2, +1.0), and caudal (AP coordinate +0.7) sites in experiment 1. Circles (O) and crosses (+) indicate loci of injector tips in the inactivation and saline groups respectively. ***B***, Locations of injector tips in the NAc intermediate/caudal core for experiment 2. Based on the stereotaxic atlas of the rat brain of Paxinos and Watson (1998). ***C***, Schematic representation of the radial distance (0.2-0.4 mm) from the injector site, in which c-Fos expression was significantly suppressed in rats microinjected with muscimol/baclofen, compared with saline control rats (***p < 0.001 at 0.2 mm, and *p < 0.05 at 0.3 mm). The photographs on the far right show representative images of c-Fos expression in the caudal NAc core of a saline-infused (top) or muscimol/baclofen-infused (bottom) animal (40× magnification).

Brain slices were verified for the placement of injector tips in the NAc core (Fig. 2), with reference to the stereotaxic atlas of the rat brain of Paxinos and Watson (1998). Data from five animals were excluded from statistical analyses due to incorrect cannula placement (*n* = 3 falling either in the shell compartment or overlying dorsal striatum), or the area of infusion having sustained substantial damage (*n* = 2). Two animals developed sickness around three weeks after surgery and did not complete the cue valence acquisition training. Furthermore, a total of eight animals (across the two experiments) demonstrated neither learning of the appetitive cue, nor the aversive cue, and were therefore excluded from further participation in the studies. The final group numbers for experiment 1 (conflict test) were: caudal core inactivation, *n* = 7; caudal core saline, *n* = 7; intermediate core inactivation, *n* = 6, intermediate core saline, *n* = 5; rostral core inactivation, *n* = 8; rostral core saline, *n* = 7. The final group numbers for experiment 2 (separate valence tests) were: intermediate-caudal core inactivation, *n* = 9 and intermediate-caudal core saline, *n* = 11.

### c-Fos characterization of spread of drug effects

Baclofen/muscimol microinfusions produced localized inhibition in the expression of the immediate early gene c-Fos, reflecting the spatial range of impact of the GABAR agonists on brain activity (Fig. 2*C*). Within a radius of 0.1 mm from the injection site, tissue damage induced marked autofluorescence, rendering the quantification of c-Fos unreliable, and thus this region was excluded from the analysis. Significant inhibition of c-Fos expression, as compared with saline controls, was found at up to 0.3-mm radial distance from the injection site (drug × distance interaction: *F*_(4,24)_ = 7.14, *p* = 0.001^°^, up to 0.2-mm radial distance, drug effect: *F*_(1,7)_ = 75.8, *p* < 0.001^q^, up to 0.3-mm radial distance: *F*_(1,7)_ = 7.11, *p* = 0.037^q^, 0.4-mm radial distance: *F*_(1,7)_ = 0.55, *p* = 0.49^q^, Table 1). An area with an average inhibition of 65.2% was found up to 0.2-mm radial distance, and a surrounding area at up to 0.3-mm radial distance was found to exhibit a 31.7% inhibition in expression. Importantly, these results give us a high degree of confidence that the inactivation effect was contained within the NAc core.

### Experiment 1 (Table 1)

#### Cue valence acquisition

**Figure 3.**
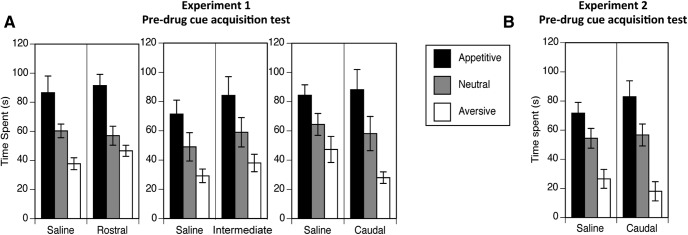
Conditioned cue preference/avoidance tests, expressed as the time spent in the appetitive, aversive, and neutral arms (±SEM), were conducted to assess the acquisition of cue valence in experiments 1 (***A***) and 2 (***B***). In both experiments, animals spent a greater amount of time in the appetitive versus the neutral cued arm and less time in the aversive as compared with the neutral arm.

A conditioned cue preference/avoidance test was conducted at the end of cue valance training to determine whether the animals had learned to associate the valenced cues with aversive and appetitive outcomes. Three-way repeated measures ANOVA of exploration time in the three maze arms (appetitive, aversive, and neutral) revealed a significant main effect of arm valence (*F*_(1.51,51.72)_ = 24.47, *p* = 0.0001^a^), indicating successful acquisition of cue valence (Fig. 3*A*). Planned comparisons of the amount of time spent exploring the appetitive and aversive arms relative to the time spent exploring the neutral arm revealed that animals spent a greater amount of time in the appetitive versus the neutral cued arm (*F*_(1,80)_ = 21.65, *p* = 0.0001^b^) and less time in the aversive as compared with the neutral arm (*F*_(1,80)_ = 9.50, *p* = 0.01^b^). No significant main effects were found for cannula location (*F*_(2,34)_ = 2.05, *p* = 0.14^a^) or drug group (*F*_(1,34)_ = 0.81, *p* = 0.38^a^), nor were there any significant interactions^a^, indicating that the groups did not exhibit any preexisting differences in cue valence learning. The time spent in the central hub compartment was also subjected to univariate ANOVA and revealed no significant differences between groups (drug group, *F*_(1,34)_ = 0.81, *p* = 0.38^c^; location, *F*_(2,34)_ =2.05, *p* = 0.14^c^; drug × cannula location interaction, *F*_(2,34)_ = 0.94, *p* = 0.40^c^).

#### Conflict test

**Figure 4.**
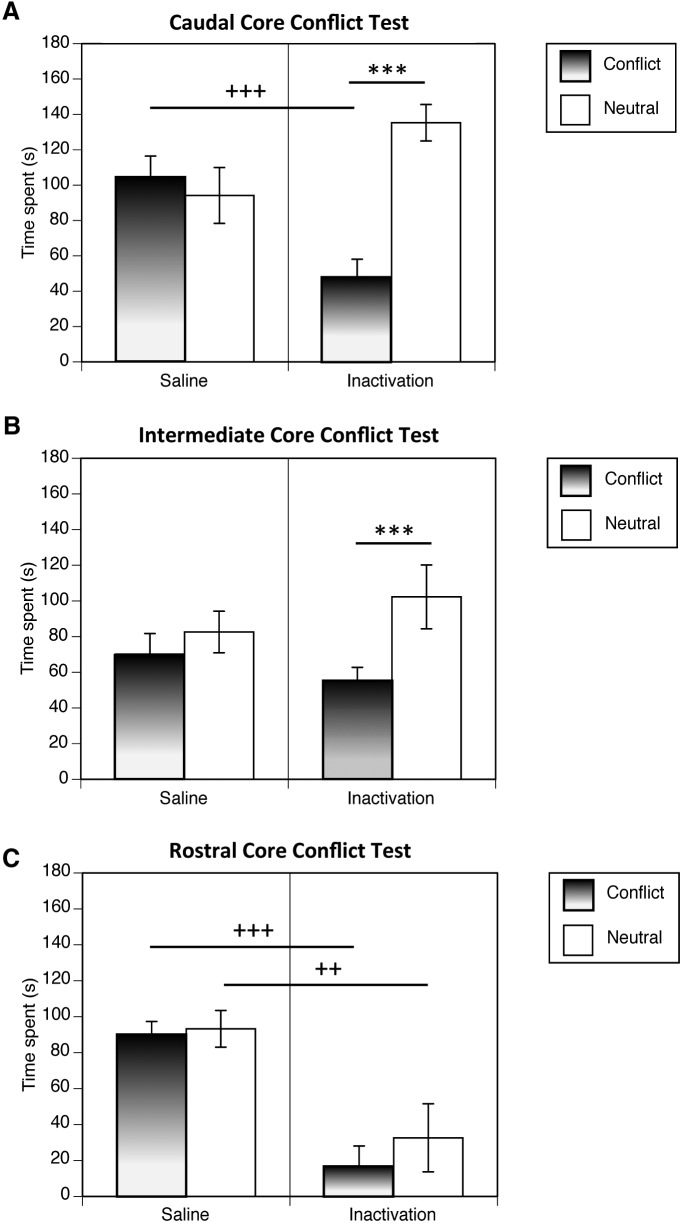
Conflict tests, expressed as the mean time spent in the arm with the superimposed appetitive and aversive cues (conflict arm), and arm with the neutral cues (±SEM), in animals undergoing caudal (***A***), intermediate (***B***), or rostral (***C***) core inactivation, or control saline-vehicle infusions. Saline-infused animals and rostral core-inactivated animals did not differ significantly in the time spent exploring the conflict versus neutral arms, whereas the caudal and intermediate core-inactivated animals spent significantly less time in the conflict as opposed to the neutral arm (***p = 0.001). There were significant differences between the time spent in the conflict arm between the saline-infused and caudal core-inactivated animals (^+++^ *p* = 0.0001) and between the saline-infused and rostral core-inactivated animals (^++^ *p* = 0.01).

A three-way repeated measures ANOVA was conducted to compare the amount of time spent in the neutral versus conflict arm for the two different drug groups (GABA receptor agonist inactivation and saline) and three different cannula locations (rostral, intermediate versus caudal accumbens core). The results indicated a main effect of arm valence on exploration time, with animals overall spending a greater amount of time in the neutral as compared with the conflict arm (*F*_(1,34)_ = 25.57, *p* = 0.00001^d^). However, there were significant differences in the degree to which different drug treatment and cannula location groups avoided the conflict arm, as revealed by a significant arm × drug group × cannula location interaction (*F*_(2,34)_ = 12.99, *p* = 0.0001^d^) and a significant interaction between arm valence and drug group (*F*_(1,34)_ = 39.35, *p* = 0.0001^d^). Tests of simple effects were conducted to further investigate the significant interactions. Among animals having cannula placements in the caudal core, inactivated animals spent significantly less time in the conflict as opposed to the neutral arm (*F*_(1,34)_ = 47.16, *p* = 0.0001^e^), while the saline-infused animals did not differ significantly in the time spent exploring the conflict versus neutral arms (*F*_(1,34)_ = 0.69, *p* = 0.41^e^) (Fig. 4*A*). In addition, there was a significant main effect of drug group for the time spent exploring the conflict arm between the caudal core-inactivated and saline groups (*F*_(1,34)_ = 15.80, *p* = 0.0001^f^). Among animals having cannula placements in the intermediate core (Fig. 4*B*), the inactivation group spent more time in the neutral as opposed to the conflict arm (*F*_(1,34)_ = 15.89, *p* = 0.0001^e^), but the saline group again did not differ the in time spent exploring the conflict versus neutral arms (*F*_(1,34)_ = 0.67, *p* = 0.42^e^). However, the time spent exploring the conflict and neutral arms in the intermediate core-inactivated group was not significantly different from that of the saline control group (conflict arm, *F*_(1,34)_ = 0.13, *p* = 0.72^f^; neutral arm, *F*_(1,34)_ = 2.42, *p* = 0.13^f^). Thus, the effect of caudal core inactivation on the expression of conflict behavior was significantly more robust than that induced by of intermediate core inactivation.

Among animals having cannula placements in the rostral core (Fig. 4*C*), neither the saline group nor inactivation group spent more time in the neutral as opposed to the conflict arm (*F*_(1,34)_ = 1.79, *p* = 0.19^e^ and *F*_(1,34)_ = 0.078, *p* = 0.78^e^). The rostral core inactivation group also showed significantly lower levels of time spent exploring both the conflict and neutral arms compared with the saline group (conflict arm, *F*_(1,34)_ = 27.58, *p* = 0.0001^f^; neutral arm, *F*_(1,34)_ = 9.02, *p* = 0.01^f^). Close observation of the behavior of the animals during the conflict test suggested a qualitative difference contributing to the decrement in total exploration time in the rostral core-inactivated animals. It was observed that these animals spent a significant amount of time performing unusual chewing behavior. Many of the animals did not exit the central hub and instead spent the duration of the testing period performing chewing motions with either no substrate in their mouths or directed toward substrates not seen in the control animals, such the edge of the apparatus or their own body parts. The nondeparture from the central hub was reflected in the results of an ANOVA conducted on the time spent in the central hub during the conflict test, which revealed that the rostral core-inactivated animals (and no other group) spent significantly increased time in the central hub compared with the saline control group (drug group × cannula location interaction, *F*_(2,34)_ = 7.16, *p* < 0.01^g^; simple effect of drug in rostral core, *F*_(1,34)_ = 19.94, *p* < 0.0001^g^; in intermediate core, *F*_(1,34)_ = 0.78, *p* = 0.39^g^; in caudal core, *F*_(1,34)_ = 0.27, *p* = 0.61^g^). Among animals that departed the central hub, some of the animals made only one arm entry in total and spent the majority of time in one place chewing on a cue, edge of apparatus, or body part. This chewing time was subtracted from the arm exploration times used in the analysis. Chewing time in the central hub was not quantified due to visibility limitations.

### Experiment 2 (Table 1)

#### Cue valence acquisition

Two-way ANOVA of the data generated from the conditioned cue preference/avoidance test revealed a significant main effect of arm valence (*F*_(2,36)_ = 26.14, *p* = 0.0001^h^). Planned comparisons revealed that animals spent a greater amount of time in the appetitive versus the neutral cued arm (*F*_(1,38)_ = 6.42, *p* = 0.02^i^) and less time in the aversive as compared with the neutral arm (*F*_(1,38)_ = 19.24*, p* = 0.0001^i^; Fig. 3*B*). There were no significant main effects found for drug group (*F*_(1,18)_ = 0.10, *p* = 0.76^h^), nor was there a significant interaction between drug group and arm valence (*F*_(2,36)_ = 1.19, *p* = 0.32^h^), indicating the absence of preexisting differences in cue valence learning (Fig. 3*B*).

#### Separate valence cue preference/avoidance tests

**Figure 5.**
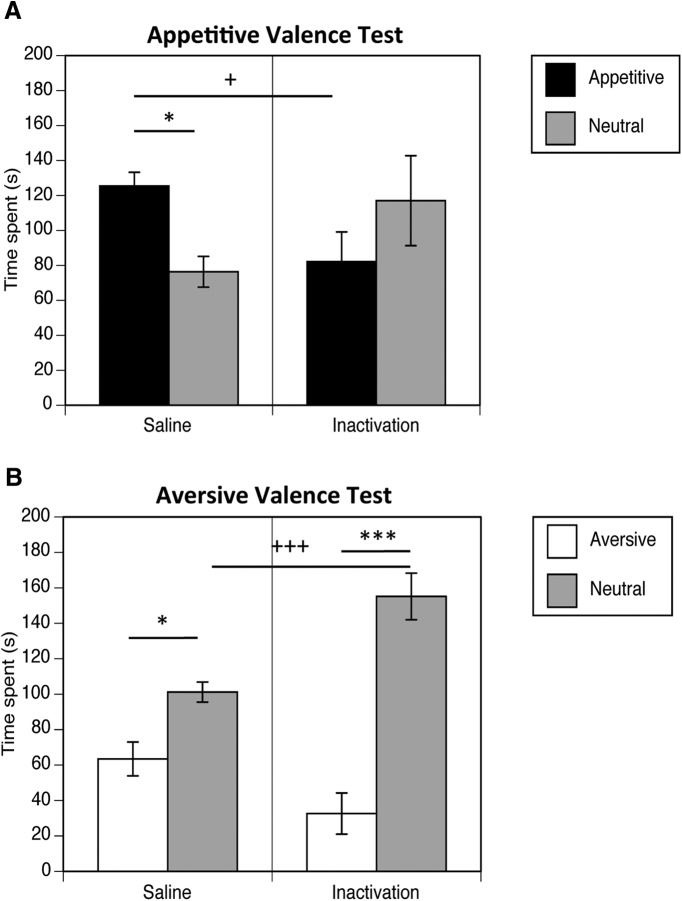
Separate appetitive (***A***) and aversive (***B***) valence tests, expressed as the mean time spent (±SEM) in the arm with the appetitive or aversive cues, versus arm with the neutral cue, were administered in animals undergoing inactivation in the caudal core, or saline vehicle infusion. Caudal core-inactivated rats failed to exhibit conditioned preference for the appetiitve cue, and displayed a decrease in exploratory time in the appetitive arm as compared with saline animals (*p = 0.05, ^+^*p* = 0.05). Furthermore, caudal core-inactivated animals exhibited potentiated conditioned avoidance of the aversive cue, and spent increased time in the neutral arm as compared with the saline group (^+++^*p* = 0.001, ***p = 0.01, *p = 0.05).

Animals were tested for the effects of caudal core GABAR antagonism on exploratory preference or avoidance in response to cues signaling appetitive or aversive outcomes in separate tests (Fig. 5). Repeated measures ANOVAs were conducted to analyze the amount of time spent in the appetitive versus neutral arms and in the aversive versus neutral arms as compared by drug group. ANOVA of the appetitive valence test data (Fig. 5*A*) indicated no significant main effect of arm valence (*F*_(1,18)_ = 0.17, *p* = 0.69^j^) or drug group (*F*_(1,18)_ = 0.01, *p* = 0.92^j^), but a significant interaction between drug group and arm valence (*F*_(1,18)_ = 5.78, *p* = 0.03^j^). Tests of simple effects attributed the significant interaction effect to be due to the inactivation group failing to show a preference for the appetitive arm over the neutral arm (*F*_(1,18)_ = 1.81, *p* = 0.20^k^), in contrast to the saline group exhibiting preference for the appetitive arm (*F*_(1,18)_ = 4.42, *p* = 0.05^k^). Furthermore, the inactivation group showed significantly decreased exploratory time in the appetitive arm as compared with saline animals (*F*_(1,18)_ = 6.05, *p* = 0.03^k^), whereas exploration time in the neutral arm did not differ significantly between drug groups (*F*_(1,18)_ = 2.63, *p* = 0.12^k^).

ANOVA of the aversive valence test data (Fig. 5*B*) revealed a significant main effect of arm valence (*F*_(1,18)_ = 49.81, *p* = 0.0001^l^), no significant main effect of drug group (*F*_(1,18)_ = 1.88, *p* = 0.19^l^), but a significant interaction between drug group and arm valence (*F*_(1,18)_ = 13.96, *p* = 0.01^l^). Tests of simple effects indicated that both the inactivated and saline groups spent significantly less times in the aversively valenced arm, compared with the neutral arm (inactivation, *F*_(1,18)_ = 52.96, *p* = 0.0001^m^; saline, *F*_(1,18)_ = 6.13*, p* = 0.03^m^). However, the inactivated animals exhibited significantly increased exploratory time in the neutral arm compared with the saline group (*F*_(1,18)_ = 16.30, *p* = 0.001^m^), coupled with lower exploratory time in the aversively valenced arm that approaches significance, as compared with saline animals (*F*_(1,18)_ = 4.29, *p* = 0.05^m^). Thus, these data indicate both a decrease in exploratory approach motivation in response to the appetitively conditioned cue, and an increased avoidance of the aversively conditioned cue in animals undergoing disruption of activity in the caudal NAc core.

#### Elevated plus maze

**Figure 6.**
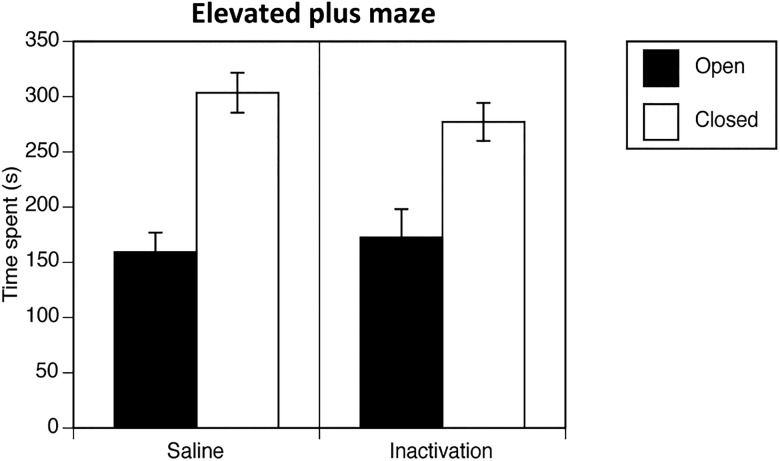
Performance of elevated plus maze, expressed as the mean time spent (±SEM) in the open and closed arms in rats undergoing inactivation in the caudal core, or saline vehicle infusion.

A significant main effect of arm was found for exploration time in the maze arms (*F*_(1,18)_ = 24.93, *p* = 0.0001^n^), indicating that all rats spent significantly more time in the closed or “safe” arm versus the open or anxiogenic arm in both the saline group and in the group undergoing inactivation in the intermediate-caudal NAc core (Fig. 6). Indeed, there was no significant main effect of drug group (*F*_(1,18)_ = 0.27, *p* = 0.61^n^) nor any significant interaction found between arm and drug group (*F*_(1,18)_ = 0.64, *p* = 0.44^n^). Thus, the drug manipulation did not significantly affect anxiety levels.

## Discussion

The present results provide evidence of a role for the intermediate to caudal NAc core in the processing of valenced cues in motivating approach and avoidance responses. The augmentation in avoidance behavior in response to cues of conflicting valence in the rats that received local GABA_A_ and GABA_B_ receptor agonism in the more caudal, but not rostral aspects of the NAc core, along with the observation that this manipulation modulated both conditioned cue avoidance and conditioned cue approach behavior, suggests that this region may have the capacity to exert differential control over cued approach and avoidance behavior. Furthermore, the elevated plus maze performance of our caudal NAc core-inactivated rats did not differ from that of the control saline group, ruling out alterations in anxiety levels as an explanation for the augmentation in the expression of avoidance behavior.

### Role of caudal NAc core in approach avoidance decision making

The emergence of a marked avoidance bias in response to the simultaneous presentation of cues of opposing valence following post-acquisition inactivation of more caudal sites in the NAc core in the present study implicates this region in the regulation of approach/avoidance behavior in the face of a learned approach/avoidance conflict. Furthermore, the observed impairment in conditioned cue preference, and contrasting enhancement of conditioned cue avoidance in caudal core-inactivated rats, suggests that the bias toward avoidance behavior observed during the approach/avoidance conflict test may have been a composite of a decreased rewarding impact of the appetitive cue, and an increased aversion of the aversive cue. Thus, the present results suggest that the NAc intermediate-caudal core region is part of a neural circuitry that has the capacity to elicit approach behavior as well as inhibit avoidance behavior in response to prevailing circumstances, as informed by behaviorally relevant cues. Such a role would be consistent with the postulated function of the NAc as a site of integration of information and a selector of appropriate behavior (Humphries and Prescott, 2010).

The notion that the NAc intermediate-caudal core serves to promote cue-elicited approach behaviors is in accord with a number of previous studies, which demonstrated disruptions in the ability of conditioned (Pavlovian) appetitive cues to elicit approach behavior as a result of NAc core manipulations (Parkinson et al., 1999, 2000; Ito et al., 2008). The dopaminergic innervation of the NAc core is also highly responsive to the presentation of reward- or drug-associated Pavlovian cues (Ito et al., 2000; Roitman et al., 2004; Saunders and Robinson, 2012). Together with further evidence that NAc core lesions abolish the ability of Pavlovian cues to invigorate an instrumental lever press response (Hall et al., 2001; Corbit and Balleine, 2011), the present results strongly implicate the more caudal core region in mediating the motivational effects of appetitive pavlovian cues, which, in turn, can elicit and/or invigorate goal-directed behavior.

The present study also supports the growing number of studies implicating the NAc in subserving aversive motivational processing (Carlezon and Thomas, 2009; Levita et al., 2009; McCutcheon et al., 2012), albeit our NAc caudal core manipulation did not disrupt avoidance behavior in the direction that may have been expected (i.e., impairment; De Leonibus et al., 2003). Instead, post-acquisition inactivation of the NAc caudal core caused a prominent augmentation in the expression of avoidance behavior in the face of an approach/avoidance conflict, as well as in conditioned cue avoidance, raising the strong possibility that the caudal core, under normal circumstances, plays a role in actively regulating cued avoidance behavior. To our knowledge, our study presents the first demonstration of a potentiation of passive avoidance behavior in response to cues predictive of shock as a consequence of disrupting NAc core function. However, there has also been a recent report of excitotoxic lesions of the NAc core resulting in an improved acquisition and retention of active avoidance in which rodents learnt to shuttle between two compartments in response to an auditory tone predictive of shock (Lichtenberg et al., 2014). Taken together, it is possible that the NAc caudal core region may play a part in actively opposing cue-elicited avoidance under certain circumstances. Converging animal and human studies have identified brain areas such as the ventral hippocampus, dorsal striatum, and NAc shell to be important in facilitating avoidance behavior (or inhibiting approach behavior - Kirkby and Kimble, 1968; Mitcham and Thomas, 1972, Allen and Davison, 1973; Viaud and White, 1989; Reynolds and Berridge, 2002; Levita et al., 2012; Palminteri et al., 2012; Schumacher et al., 2016), providing potential sites on which the NAc caudal core may be able to exert its inhibition. For instance, the NAc caudal core is able to exert inhibitory control over the dorsal striatum, via direct medium spiny (GABAergic) projections or indirectly via striatal-nigro-striatal dopamine pathways (Haber et al., 2000). Furthermore, the NAc caudal core has the means to modulate NAc shell activity via medium spiny neurons (MSNs-GABAergic) collateral projections that span the entire extent of the shell (Van Dongen et al., 2005). Further work will need to be conducted to investigate these possibilities.

### Rostrocaudal gradient of bivalent motivational processing in NAc core

Previous studies involving pharmacological manipulations of the NAc shell found opposite effects of inactivation in the caudal versus rostral regions in the elicitation of negatively versus positively valenced consummatory and defensive behaviors (Reynolds and Berridge, 2001; Faure et al., 2008). A similar assessment in the NAc core [using glutamate (AMPA) receptor antagonism] did not demonstrate any difference in these innate reactions to unconditioned stimuli along a rostrocaudal gradient, suggesting that the core may not exhibit the same regional bivalent gradation in valence processing (Reynolds and Berridge, 2003). The results of the present study now extend these findings with the use of GABA (A and B) receptor agonists and suggest that conditioned responses elicited by valenced cues are not differentially represented along the rostrocaudal gradient of the NAc core. Focal GABA_A_ and GABA_B_ receptor agonism in the intermediate to caudal core in the present study led to a potentiated expression of conditioned avoidance behavior, closely mirroring the elicitation of avoidance and unconditioned fear behaviors following NAc caudal shell GABA_A_ receptor activation (Reynolds and Berridge, 2002). However, we did not find any evidence of a potentiation in cue-induced approach behavior as a result of rostral core inactivation such as that previously seen after inactivation of rostral shell sites (Faure, et al., 2008; Reynolds and Berridge, 2001). That is, the rostral core-inactivated animals in the present study did not increase their motivation to approach the appetitive cue above control levels. However, one limitation in the interpretation of the current behavioral results with reference to a rostrocaudal gradient in approach and avoidance is the qualitatively different nature of the behavior of the rostrally versus caudally inactivated animals. The former animals displayed lower total exploration times, which appeared to be largely mediated by compulsive chewing behavior. Of particular note was the fact that the chewing occurred indiscriminately, on any object or surface that was placed in front of the rats, reminiscent of the manifestation of vacuous chewing in animal models of tardive dyskinesia (Kulkarni and Naidu, 2001), a disorder of involuntary repetitive movements that can develop in patients undergoing long-term treatment with dopamine antagonists commonly used as antipsychotics medications.

### Estimation of drug spread using c-Fos immunohistochemistry

In the present study, the disruption of local neural circuits in the NAc core was achieved by the powerful combination of activating GABA_A_ (located on proximal dendrites and somata of the MSNs) and GABA_B_ receptor (located in the terminals of glutamatergic afferents to the MSNs) receptors, which was accompanied by a radial area of c-Fos suppression up to 0.3 mm around the infusion site. This may seem like a modest estimation of drug spread, when compared with the results of previous studies that have used visualizable dye to estimate the spread of muscimol (but not combination of muscimol and baclofen). For instance, Lohman et al. (2005) injected 0.2-μl of methylene blue into the thalamus and observed a radial spread of ∼0.5 mm. However, we would argue that there are caveats to the interpretation of dye spread as a proxy for effective drug spread. The spread of individual substances varies based on the volume and electrostatic properties of a given compound, and even different dyes have been found to exhibit differential spreads (Myers, 1966). Additionally, the visualizability of a dye at a given distance does not necessitate that the concentration of a drug at that distance would be sufficient to have a significant biological effect, which is important considering that the effect of suppression by muscimol is lessened with greater distance and corollary lower concentration. Crucially, the estimated spread of the combined muscimol/baclofen in the present study using c-Fos expression, a powerful marker of neuronal activity (Bullitt, 1990), is more consistent with the findings of studies that have used similar approaches. Faure et al. (2010) used a volume of 0.5-μl muscimol alone and found a very focal attenuation in c-Fos activity up to a 0.3-mm radius, which was accompanied by a marked differentiation in the processing of motivational valence and hedonic information along the rostrocaudal gradient of the NAc shell. Furthermore, Arikan et al. (2002) used a combination of electrophysiology and light microscopy to assess the effective spread of 1 μl of 2% muscimol and found that neural activity was absent in a region (cerebellum) up to a 1-mm radius from the injection site. Assuming that the extent of diffusion correlates with the injected volume, the effective spread of 0.3 mm that we have observed with a microinjection of 0.3-μl muscimol/baclofen in the present study is highly consistent with the degree of spread that Arikan et al. (2002) would predict.

In conclusion, the present study implicates the more caudal aspect of the NAc core to be critical in the regulation of approach avoidance behaviors in response to valenced cues. More specifically, the activation of caudal core may preferentially facilitate the elicitation of approach behavior, and suppress avoidance behavior in situations in which an encounter with ambivalent/bivalent cues may force approach/avoidance decisions. Together with electrophysiological evidence that separate neuronal populations coding the values of cues associated with reward versus punishment are colocalized in discrete pockets within the NAc (Roitman et al., 2005), the present study suggests that the caudal region of the NAc core is potentially the site at which the motivational impact of valenced cues is integrated to elicit approach behavior. Furthermore, the varying behaviors elicited by the manipulation of the caudal and rostral subregions of the NAc core is coherent with the concept that this structure should be considered as a composite of distinguishable neuronal ensembles, as opposed to a homogeneous structure with a monolithic function and output (Pennartz, et al., 1994), serving to highlight the necessity of carefully targeting multiple coordinate sites along the rostrocaudal axis in the determination of the functions of the NAc core. Further research needs to be directed at elucidating the neural network that the caudal core is a part of, in the representation and processing of cue valence and its impact on motivated behavior.

## Synthesis

Reviewing Editor: Philippe Tobler, University of Zurich Decisions are customarily a result of the Reviewing Editor and the peer reviewers coming together and discussing their recommendations until a consensus is reached. When revisions are invited, a fact-based synthesis statement explaining their decision and outlining what is needed to prepare a revision will be listed below. The following reviewer(s) agreed to reveal their identity: James McCutcheon, Vedran Lovic

This manuscript describes a series of experiments that look at the behavioral responses to inactivation of different rostrocaudal sites within nucleus accumbens core. In particular, the focus is on a task involving conflict between cues that have been conditioned with opposing valences. Microinfusions of GABA receptor agonists are used to perform localized and reversible inactivations. The main finding are (1) that inactivations cause an increase in avoidance of the maze arm that contains conflicting cues in preference for a neutral arm and (2) that this effect is graded along the rostro caudal axis with greater effects seen in caudal core relative to rostral core. Furthermore, inactivation lead to a decrease in approach towards positively valenced arms and enhancement of avoidance of negatively valenced arms.

Major points

1 The finding that is least supported is the differentiation between rostral and caudal sites, as described by the authors. This interpretation is severely compromised by the effects observed when rostral core is inactivated, which include a large decrease in general exploration. The way in which the correlation is then computed using a difference score and not taking into account this drastic change in behavior means that the conclusions are tenuous at best. As it stands, this assertion in the paper will have to be removed unless better supported:

a. The difference in exploration in the rostral core inactivation group needs to be better described in the Results. In the Discussion the qualitatively different nature of drug effect is explained as being an increase in non-specific chewing etc. This needs to be highlighted in the Results.

b. Given the above, calculation of a difference score does not seem the most appropriate way of performing the regression. Does the regression still hold up if a preference score - which would be normalized to total exploration time - is used? For example, (time in conflict arm)/(time in conflict arm - time in neutral arm)

2 Please provide more information on the USs used. Sucrose solutions and foot shocks have different sensory modalities - taste vs. tactile/somatosensory. How were the intensities chosen? There should be some information about their equivalencies (or lack there of). Is 2ml of 20% sucrose as appetitive as a 0.25mA shock is aversive? Do you have data that speak to this or can you otherwise comment on this in the manuscript?

3 The reviewers were somewhat surprised at how focal and small the locus of inhibition with muscimol/baclofen combination was, at least based on Fos immunohistochemistry. It seems the manipulation produced inhibited only a fraction of NAc core.

a. Using Fos as the sole measure of inhibition localization may underestimate diffusion and a comparison with previously published dye measures should be provided in the discussion.

b. An image of c-Fos staining should be included.

4 It might be useful to strengthen the rationale for doing these types of experiments. Real life situations rarely involve responses towards pure positively or negatively valenced stimuli. The vast majority of situations will involve conflicts. Some sentences along these lines suffice and they would make the manuscript more attractive to a non-expert reader.

5 How should non-arm time be interpreted? It seems that rats spend about ~200s in one of the arms. The test is 300s long, which suggests that ~100s is spent in the central platform. This is quite a significant amount of time. Is the central platform a neutral zone as well? If it is, would this change any of the interpretations or statistics? This should be discussed. Relatedly, is information on crossings between arms available as well as the time spent in each arm?

Minor points

1 Figure 3 is confusing. It shows preference in rats/groups prior to any drug manipulation (which is fine). However, the labels might be misleading the reader, suggesting that rats got either an infusion of saline or drug in one of 3 regions of NAc. This should somehow be fixed (e.g., a label above the graph saying “predrug/treatment preferences ”).

2 In the Introduction, the Nieh reference could be supplemented with additional, earlier citations that posit a role of this circuitry.

3 Information on the method of brain sectioning should be provided.

4 The secondary antibody should be described properly.

5 A figure should be referenced when results of cue-valence association are being described.

6 Page 13, last sentence of the second paragraph: “media-caudal core inactivation ” should be changed to “medial-caudal core inactivation ”.

7 On page 17, the phrase “an increase in avoidant exploration ” is not clear.

Further note (no need for changes)

This type of project would have benefited from the use of chemogenetic/DREADD approaches. By using DREADD approaches, greater areas of rostral, medial, and caudal NAc could have been inhibited (but see major point 2). Furthermore, inhibition, via single i.p. injection of clozapine N-oxide, would have been far less stressful for the rats.
